# Integrating animal movements with phylogeography to model the spread of PRRSV in the USA

**DOI:** 10.1093/ve/veab060

**Published:** 2021-07-15

**Authors:** Dennis N Makau, Moh A Alkhamis, Igor a. D Paploski, Cesar A Corzo, Samantha Lycett, Kimberly VanderWaal

**Affiliations:** Department of Veterinary Population Medicine, College of Veterinary Medicine, University of Minnesota, Minneapolis, 1365 Gortner Avenue, St. Paul, MN, 55108, USA; Department of Epidemiology and Biostatistics, Faculty of Public Health, Health Sciences Center, Kuwait University, Kuwait City, 24923, Safat 13110, Kuwait; Department of Veterinary Population Medicine, College of Veterinary Medicine, University of Minnesota, Minneapolis, 1365 Gortner Avenue, St. Paul, MN, 55108, USA; Department of Veterinary Population Medicine, College of Veterinary Medicine, University of Minnesota, Minneapolis, 1365 Gortner Avenue, St. Paul, MN, 55108, USA; Roslin Institute, University of Edinburgh, Edinburgh, Midlothian, EH25 9RG, UK; Department of Veterinary Population Medicine, College of Veterinary Medicine, University of Minnesota, Minneapolis, 1365 Gortner Avenue, St. Paul, MN, 55108, USA

**Keywords:** Bayesian inference, phylodynamic models, phylogeography, molecular epidemiology, animal movement, livestock networks

## Abstract

Viral sequence data coupled with phylodynamic models have become instrumental in investigating the outbreaks of human and animal diseases, and the incorporation of the hypothesized drivers of pathogen spread can enhance the interpretation from phylodynamic inference. Integrating animal movement data with phylodynamics allows us to quantify the extent to which the spatial diffusion of a pathogen is influenced by animal movements and contrast the relative importance of different types of movements in shaping pathogen distribution. We combine animal movement, spatial, and environmental data in a Bayesian phylodynamic framework to explain the spatial diffusion and evolutionary trends of a rapidly spreading sub-lineage (denoted L1A) of porcine reproductive and respiratory syndrome virus (PRRSV) Type 2 from 2014 to 2017. PRRSV is the most important endemic pathogen affecting pigs in the USA, and this particular virulent sub-lineage emerged in 2014 and continues to be the dominant lineage in the US swine industry to date. Data included 984 open reading frame 5 (ORF5) PRRSV L1A sequences obtained from two production systems in a swine-dense production region (∼85,000 mi^2^) in the USA between 2014 and 2017. The study area was divided into sectors for which model covariates were summarized, and animal movement data between each sector were summarized by age class (wean: 3–4 weeks; feeder: 8–25 weeks; breeding: ≥21 weeks). We implemented a discrete-space phylogeographic generalized linear model using Bayesian evolutionary analysis by sampling trees (BEAST) to infer factors associated with variability in between-sector diffusion rates of PRRSV L1A. We found that between-sector spread was enhanced by the movement of feeder pigs, spatial adjacency of sectors, and farm density in the destination sector. The PRRSV L1A strain was introduced in the study area in early 2013, and genetic diversity and effective population size peaked in 2015 before fluctuating seasonally (peaking during the summer months). Our study underscores the importance of animal movements and shows, for the first time, that the movement of feeder pigs (8–25 weeks old) shaped the spatial patterns of PRRSV spread much more strongly than the movements of other age classes of pigs. The inclusion of movement data into phylodynamic models as done in this analysis may enhance our ability to identify crucial pathways of disease spread that can be targeted to mitigate the spatial spread of infectious human and animal pathogens.

## 1. Introduction

Porcine reproductive and respiratory syndrome (PRRS) is a viral disease of swine caused by a ribonucleic acid (RNA) arterivirus broadly classified as PRRS virus (PRRSV) Type 1 (*Eurpobartevirus Betaarterivirus suid 1*) and Type 2 (*Ampobartevirus Betaarterivirus suid 2*) (Kuhn et al., 2016; Shi et al., 2010a; Stadejek et al., 2013; Walker et al., 2020). PRRS is arguably the most expensive swine disease in the USA, with annual economic losses estimated at ∼$664 million/year (Holtkamp et al., 2013; Pileri and Mateu 2016; Nathues et al., 2017). These losses are associated with decreased reproductive performance and piglet mortality, although the severity of clinical disease varies with the infective strain of PRRSV and other farm management practices (Goldberg et al., 2000).

Since the initial detection of PRRS in the USA in the early 1990s, the swine industry has made considerable efforts to understand and manage the disease. These efforts have included the implementation of strict biosecurity measures on swine farms (Velasova et al., 2012; Silva et al., 2019), different immunization programs (Corzo et al., 2010), disease surveillance, and monitoring programs (Perez et al., 2019) among others. Despite these efforts, effective management and control of PRRS remain a challenge. These difficulties have been attributed to several factors, key among them being the importance of animal movements and environmental factors in between-farm spread (Otake et al., 2010; Pileri and Mateu 2016; Arruda et al., 2018a; VanderWaal et al., 2020) and rapid viral evolution resulting in substantial genetic and antigenic diversities (Charerntantanakul 2012; Paploski et al., 2019).

PRRSV Type 2 is typically classified according to restriction fragment length polymorphisms (RFLPs) or phylogenetic relatedness (Shi et al., 2010b; Paploski et al., 2019). PRRSV Type 2 viruses were originally classified into nine phylogenetic lineages (Paploski et al., 2019; Shi et al., 2010b). Around 2014, a novel and virulent PRRSV Type 2 strain was reported in US farms, causing high piglet mortalities and severe clinical diseases in sows. This strain was classified as RFLP Type 1-7-4 (Wang et al., 2015; van Geelen et al., 2018) and belonged to a sub-lineage of lineage 1, denoted as 1A (L1A) (Shi et al., 2010b; Paploski et al., 2019). L1A became the dominant sub-lineage of PRRSV (60 per cent of the sequences reported in the region between 2014 and 2017). The effective population size of L1A increased tremendously around 2014, suggesting some changes in the epidemiological and evolutionary dynamics of the L1A sub-lineage, notwithstanding co-circulating strains (Paploski et al., 2021). The spread of this viral variant has been shown to be associated with genetic recombinations and spatial spread through animal movements (Wang et al., 2015; van Geelen et al., 2018; Ramírez et al., 2019; VanderWaal et al., 2020; Makau et al., 2021). Epidemiological analyses suggest that the occurrence of L1A and other co-circulating lineages/sub-lineages was driven more by animal movements than the spatial proximity of farms, and secondary farm contacts (via animal movements) with L1A-positive farms were a risk factor for L1A occurrence on a farm (Makau et al., 2021). That being said, local-area spread between neighboring farms cannot be ruled out (VanderWaal et al., 2020) and a farm’s risk of viral outbreaks is increased when animal movements are received by neighboring farms (Machado et al., 2019).

Using sequence data, Bayesian phylodynamic models provide a quantitative framework to test the hypotheses about viral evolution and transmission, reconstruct the geographic patterns of spread, and map the flows of population connectivity (Alkhamis et al., 2016). Recent phylodynamic approaches allow for the inclusion of an array of relevant factors that may be associated with the spatial dispersal of a phylogeny (Lemey et al., 2009, 2010, 2014; Drummond et al., 2012; Rambaut et al., 2018). Primarily, these approaches have enabled the inclusion of spatial and environmental data in phylodynamic analysis to understand the spread of different epidemics, e.g. Ebola, influenza and HIV in humans (Dudas et al., 2017; Müller, Rasmussen, and Stadler 2018; Rasmussen et al., 2018), foot-and-mouth disease in livestock (Duchatel, Bronsvoort, and Lycett 2019; Munsey et al., 2021), and infectious wildlife diseases (Fountain-Jones et al., 2017; Yang et al., 2019; Dellicour et al., 2020). Given the role of long-distance animal movements in the spread of PRRS and other livestock diseases (Mortensen et al., 2002; Carlsson et al., 2009; Amirpour et al., 2017; Neira et al., 2017; VanderWaal et al., 2020; Makau et al., 2021), the inclusion of empirical animal movement data in these models could increase the accuracy and robustness of phylodynamic inference and is essential for capturing how host population connectivity interacts with spatial drivers of transmission.

The usage of phylodynamic methods in understanding PRRS is a growing field (Shi et al., 2010a; Franzo et al., 2015; Alkhamis et al., 2016, 2017; Sun et al., 2019; Jara et al., 2020). These models have been instrumental in the identification of high-risk areas and transmission routes for epidemic management and disease surveillance in China (Sun et al., 2019) and have identified sow farms as a high-risk group facilitating the spread of PRRSV between different farm types in the USA using discrete-trait phylodynamic models (Alkhamis et al., 2016; Jara et al., 2020). Even fewer studies have utilized recently developed inferential framework to evaluate the contribution of external factors to spatial spread (Jara et al., 2020). However, none of these studies have incorporated data on pig movements into a phylogeographic framework, which limits the inference and acuity of observed conclusions related to viral dispersal.

The US swine industry is vertically integrated and consolidated into multi-site production systems, whereby different phases of the pig production lifecycle (i.e. farrowing, weaning, growing and finishing) occur at separate locations (Lee et al., 2017; Passafaro et al., 2020). Generally, the directional flow of pigs would be from sow farm to nursery and, then, to growing/ finishing sites. Multi-site pig production systems form complex networks where different farm types are connected via the movement of pigs of different ages within the production chain (Lee et al., 2017; Kinsley et al., 2019). The transportation of weaned pigs (∼3 weeks) connects sow farms to nurseries/wean-to-finish farms, while the movement of feeder pigs (8–25 weeks old) connects nurseries/wean-to-finish farms to finishing farms The transportation of replacement gilts for breeding purposes connects gilt development units or finishing farms to sow farms. These different connections between farms pose different levels of risk for disease transmission because the management styles and levels of biosecurity vary between farm types. For example, better biosecurity and prophylaxis are practiced in sow farms and gilt development units compared to the other farm types, while most nursery farms handle animals in all-in/all-out cohorts, filling and completely emptying barns of all animals at the same time as they are moved to the next phase of production (USDA 2002). Despite the outsized role of such movement networks in the spread of pathogens (Gibbens et al., 2001; Kao 2002), animal movement data are rarely incorporated into phylogeographic models of pathogen spread.

Therefore, the objective of this study was to determine factors associated with the viral evolution and spatial diffusion of PRRSV L1A in a swine-dense production area in the USA. Through integrating Bayesian phylodynamic models built with PRRSV ORF5 sequence data with empirical pig movement data, we investigate the role of host population connectivity and the relative importance of animal movements stratified by age class in the spread of PRRSV. We hypothesized that different types of animal movements have differing relative impacts on the spread of PRRSV in the study area. Given the role of animal movements in the spread of PRRSV in the USA (Shi et al., 2010b; VanderWaal et al., 2020), the inclusion of animal movement data is expected to improve our ability to interpret spatiotemporal evolutionary trends of PRRSV.

## 2. Materials and methods

### 2.1 Sequence data

Sequences obtained from two production systems within the study region, collected through the Morrison Swine Health Monitoring Project (MSHMP), were used for this analysis. MSHMP is a nation-wide swine health monitoring program based at the University of Minnesota. This program is based on voluntary self-reporting of infection status of swine herds and collates data from swine production companies, veterinarians, and regional disease control programs (Perez et al., 2019). MSHMP monitors the health status of approximately half of the US swine-breeding population. The swine-dense region that comprises our study area encompasses approximately 15 per cent of the US breeding herd, and our study includes data from ∼90 per cent of swine-breeding and grow/finish premises in this region. Participating production systems share PRRSV sequences isolated on their farms either during routine sampling or in the course of an active outbreak. Lineage identification and classification of open reading frame 5 (ORF5) was based on phylogenetic classification by Paploski et al. (2019). ORF5 is one of 10 ORFs in the PRRSV viral genome. It is ∼600 base pairs long and encodes for the main envelope protein referred to as GP5. This protein has several antigenic sites that stimulate host immune response upon infection (Dea et al., 2000; Kim et al., 2013) and has been widely used for molecular epidemiological investigations and classification of PRRSV Type 2 (Kapur et al., 1996; Wesley et al., 1998; Shi et al., 2010b; Paploski et al., 2019). Sequence assembly and preparation was done using protocols described by Paploski et al. (2019). The sequences used in this analysis are part of the dataset reported in Paploski et al. (2019) and are available in GenBank under the accession numbersMN498289-MN502669.

### 2.2 Preliminary phylogenetic analysis

A total of 1,514 L1A sequences were obtained from 651 farms in the study area, isolated between 2014 and 2017. Of these sequences, 81 were excluded from the analysis due to incomplete geographical metadata. Of the remaining 1,433 sequences, 440 were genetic duplicates (i.e. sequences that were 100 per cent genetically similar to at least one other sequence). 37 per cent of identical sequence pairs were isolated from the same farm, and 38 per cent of identical pairs belonged to the same sector. Duplicate sequences were excluded from the analysis, with only the earliest isolated sequence of the duplicate being retained in the data (Supplementary Fig. S1). The remaining 993 sequences were aligned using the MUSCLE algorithm in AliView (Larsson 2014) using default settings. We used RAxML (v8) to construct a maximum-likelihood (ML) phylogenetic tree (using the GTR + Γ substitution model within 100 bootstraps) and used TempEst (v 1.5.3) to assess the temporal signal of the ML tree (correlation coefficient = 0.5, *R*^2^ = 0.3) (Rambaut et al., 2016). Subsequently, detection of recombinants was done using the recombinant detection program (RDP v 4.1) (Martin et al., 2015), and nine potential recombinant sequences (Maxchi and 3Seq *P*-value <0.01) were also excluded from the analysis. Ultimately, we used 984 ORF5 PRRSV L1A sequences isolated from 515 farms in a swine-dense production area in the USA, between 2014 and 2017. A second ML tree constructed using GTR + Γ substitution model within 100 bootstraps in RAxML (v 8) was then assessed for temporal signal using TempEst (v 1.5.3) (correlation coefficient = 0.7, *R*^2^ = 0.4) (Rambaut et al., 2016).

### 2.3 Overview of spatial diffusion analysis

We used a discrete-space generalized linear model (GLM) implemented within a Bayesian evolutionary analysis by sampling trees (BEAST v1.10.4) framework to quantify the effects of different factors on the rate of viral transitions between different sampling locations (Lemey et al., 2014). This model framework allows for the identification of predictors that either enhance or impede the spatial dispersal of the virus through estimating their effect on the rate of viral spread and posterior inclusion probabilities in the model, evaluated on the basis of the Bayes factor (BF). We used this framework to investigate long-distance spread of the L1A at the regional level (a broader scale than farm-to-farm transmission). In this approach, the viral transition rate matrix between discrete locations is parameterized as a log-linear function for the predictive potential of hypothesized covariates included in the model. These are used in a Bayesian model and interpreted in combination with phylogeographic reconstruction to explain spatial diffusion of the virus. Model predictors can be defined as attributes of either the origin/destination (i.e. farm density, environmental/landscape attributes, etc.) or an attribute of the origin-destination dyad (i.e. spatial adjacency or pig movements between two sectors) (see below for details).

### 2.4 Geographical region classification

**Figure 1.**
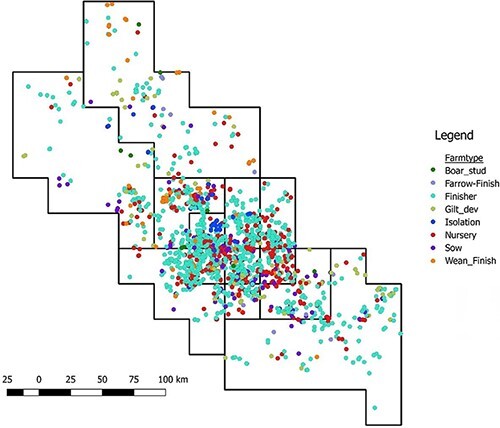
Distribution of swine farms in the 13 sectors in a swine-dense production area in the USA. The size of the sectors ranges from 436 to 5,972 mi^2^ (1,130–15,468 km^2^). Sectors vary in size in order to achieve comparable availability of data in each sector. Movement data were obtained for these farms, but not all farms provided sequence data for this analysis.

The swine-dense production region from which sequences were isolated spans ∼85,000 mi^2^ (approximately 220,000 km^2^). The study area was first subdivided into a grid of 61 sectors of equal size (∼20 × 20 mi, 436 mi^2^) using QGIS software (QGIS Development Team 2019), but some sectors only had a single sequence. Therefore, some sectors were merged to create 13 sectors in total ensuring a minimum of at least 30 sequences per sector, with the area of sectors ranging between 436 and 5,972 mi^2^ (1,130–15,468 km^2^) (Fig. 1). The risk of local introduction of viral diseases like porcine epidemic diarrhea virus into farms has been associated with attributes of the farm’s surrounding neighborhood, including movements made by neighboring farms (Machado et al., 2019). Based upon this finding, we hypothesize that the overall between-sector flow of animals (summed across all farms in the sectors) will shape patterns of dispersal of the virus across sectors, even though not all farms engaged in such movements. Summarizing the farms into sectors enabled us to account for movement-mediated connectivity of host meta-populations, as well as summarize other environmental factors that may promote transmission. The number of farms per sector ranged from 60 to 227, with 35–155 sequences recorded through time in each sector. Subsequently, all other model covariates (see below) were summarized at the sector level. A queen adjacency matrix was also created to account for the spatial structure of the population, whereby a 0/1 binary value was assigned to each matrix cell as an indicator of whether two sectors bordered one another. This matrix was included in the phylogeographic GLM as an indicator of spatial proximity between sectors. Centroids for each sector were subsequently used to visualize spatiotemporal diffusion. The average distance between sector centroids was 98.4 km with a range of 27–264 km.

### 2.5 Predictors of spatial diffusion

The between-farm spread of PRRS has been ascribed as either local-area spread or long-distance spread, the latter of which is strongly linked to animal movements (Lager, Mengeling, and Wesley 2002; Arruda et al., 2018a, 2019; VanderWaal et al., 2020). Spatial proximity, wind dispersal, and physical contamination are some of the factors largely associated with local-area spread of PRRSV (Lager, Mengeling, and Wesley 2002; Arruda et al., 2019). Land topology, vegetation cover, and land use have also been shown to correlate with risk of viral outbreaks in swine farms (Arruda et al., 2018a; Machado et al., 2019). Therefore, we included these factors in our model to account for the potential effects of these factors on the spatial diffusion of PRRSV.

The mean normalized difference vegetation index (NDVI) was summarized across a 5 km pixel raster of NDVI within the sectors. Land use data were obtained from the national land cover database (MRLC 2016). Since the database is not updated yearly, the most relevant raster file from 2016 was used to calculate the proportion of each sector that was forested vs. crop cover. This decision was guided by the assumption that changes in land use trends would be minimal given the brevity of the study period (4 years). Data on wind speed, ambient temperature, and precipitation were obtained from the National Oceanic and Atmospheric Administration (NOAA) database (NOAA 2019). The average ambient temperatures for each of the 13 sectors were calculated from mean monthly ambient temperature for the study area between 2014 and 2017. Average wind speed and mean precipitation for each sector were also calculated for the study period. Given the size of our study area, the resolution for mean ambient temperature and average wind speeds was coarse and there was minimal variation between sectors. These two predictors were excluded from the model.

Data on pig population and distribution of farms in the sectors were obtained from the MSHMP database, and farm density was calculated as a function of the number of farms in a sector and sector area (km^2^). Animal movement data were summarized in two forms. First, the directional flow of all animals shipped between farms located within any two sectors was summed to obtain the total flow of animals moving between two sectors. These total flows were stratified by the type of movements based on the production type of the origin and destination farms, which also correspond to the age class of animals moved: breeding movements (from gilt development units to sow farms, finishing farms to sow farms, finishing farms to isolation units [animals of breeding age ≥21 weeks old]), weaned pig movements (from sow farms to nursery/wean-to-finish farms [3–4 weeks old]), feeder pig movements (from nursery to finishing farms or wean-to-finish to finishing farms [8–25 weeks]), and same-phase movements (between farms of the same production level [animals of same age]). Second, farm-level metrics from a social network analysis were calculated from a static farm-level network of animal movements during the entire study period. These metrics included farm indegree (the number of farms from which a farm received animals), outdegree (the number of farms to which a farm sent animals), normalized mean betweenness (the frequency with which a farm was located on the shortest (geodesic) path between other pairs of farms), and closeness (the total geodesic distance between a farm and other farms in the network along the shortest path between them) (Wasserman and Faust 2009; Lee et al., 2017). These farm-level metrics were then averaged for farms within each sector to obtain mean sector indegree, outdegree, betweenness, and closeness. There was minimal variation in the sector mean betweenness and closeness, which were therefore excluded from the model. To account for uneven distribution of the number of sequences in the different sectors, the total number of sequences in each sector was also summarized and included as a model covariate. Ultimately, all sector covariates were arranged in a matrix (for movement data and spatial adjacency) and data frames (for other covariates) and used for subsequent modeling using BEAST.

### 2.6 Phylogeographic model

We used a relaxed-clock model and choice of tree priors to reconstruct virus population demographics in BEAST (v1.10.4) (Suchard et al., 2018). Further, we used the discrete-space GLM (Lemey et al., 2014; Magee, Suchard, and Scotch 2017) implemented in BEAST, with the predictors described above, to infer the phylogeographic diffusion process between different sectors. Specifically, we selected the general time-reversible and discrete gamma distribution models (GTR + Γ) (Felsenstein 1981; Tavare 1986) as the best-fitting substitution model using the Bayesian information criterion as implemented in PartitionFinder (v1.1) (Lanfear et al., 2012). We also evaluated different combinations of the uncorrelated lognormal (UCLN) and uncorrelated exponential branch-rate clock model and coalescent population models (Bayesian Skygrid (Sg) (Gill et al., 2013), constant population size (Kingman 1982), expansion growth (Ex), and exponential growth (Exg) (Griffiths and Tavare 1994) coalescent models) to identify the best-fitting phylodynamic model for our data out of eight candidate models. We used the marginal likelihood estimated by path-sampling (Baele et al., 2012) and stepping-stone (Fan et al., 2011) methods to select among candidate models.

We used 350 million Bayesian Markov chain Monte Carlo (MCMC) cycles and sampled every 35000th state to infer the posterior evolutionary parameters for each candidate model. Furthermore, for each model, we used duplicate MCMC runs to evaluate the stability of their marginal-likelihoods estimates. Additionally, we assessed the convergence of the posterior parameters through evaluating the effective sample size >200 using Tracer (v1.7.1) (Rambaut et al., 2018). The best-fitting phylodynamic model to the sequence data was the Sg + UCLN (BF > 400).

We summarized the resulting posterior probability density of the selected model as a maximum clade credible (MCC) tree using TreeAnotator (v1.10.4). R packages *ggtree* (Yu 2020) and *ggplot2* (Wickham 2016) were used to generate plots for the MCC tree and the effective population over time from the Skygrid coalescent model output. For GLM predictors, we used Bayesian stochastic search variable selection to identify well-supported model covariates explaining the phylogenetic diffusion (BF estimates) and posterior probabilities of inclusion. We also summarized their contribution to the log-linear rate matrix to calculate the coefficient estimate of the model predictor on viral spread given their inclusion in the model. SpreaD3 (v0.9.7.1) (Bielejec et al., 2016) was used to generate a spatiotemporal distribution map by combining geographical data using sector centroids as reference points and the best phylogenetic tree output.

## 3. Results

A total of 984 ORF-5 sequences, with a mean nucleotide identity of 98.3 per cent (standard error 0.2 per cent), from 515 farms were used in this analysis. Most of the sequences (45.6 per cent) were obtained from sow farms and gilt development units, 17.2 per cent from nursery and 14.0 per cent from finisher farms (Supplementary Fig. S1). Out of the 14 factors intended for inclusion in our model, two factors were excluded during model parameterization. We excluded the same-phase movements, which were rare (occurred only in 2014). Total pig flow between sectors was strongly correlated with weaned and feeder pig flows between sectors (Spearman’s correlation 0.9,0.8 [*P* < 0.01], respectively) and therefore were also excluded.

### 3.1 Phylogeographic analysis

**Figure 2.**
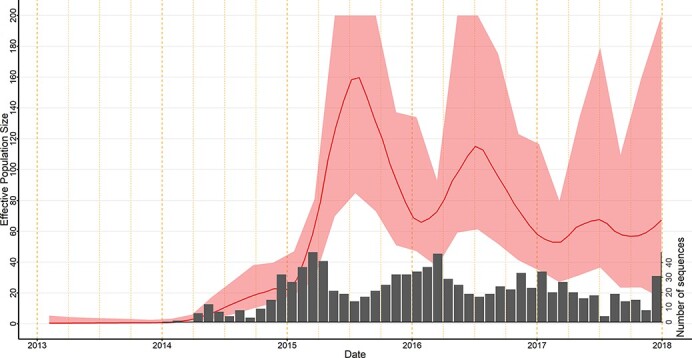
Temporal distribution of PRRSV sub-lineage 1A (L1A) in a swine-dense production area in the USA, between 2013 and 2017.The gray bars represent the frequency of sequences obtained monthly between 2014 and 2017.

**Figure 3.**
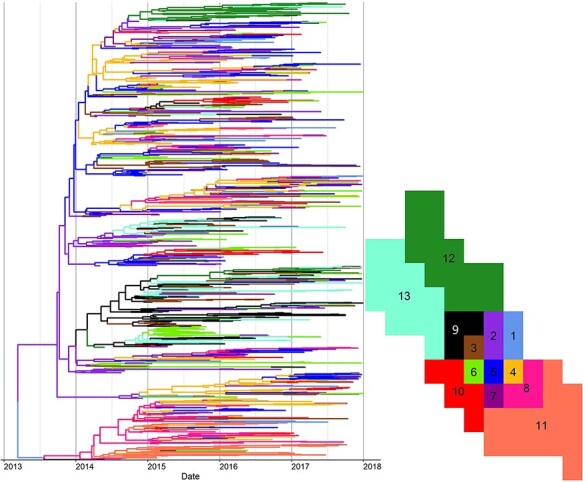
Bayesian maximum clade credibility time-scaled phylogenetic tree for PRRSV sub-lineage 1A (L1A) between 2013 and 2017. Branches are colored according to their inferred sector of origin, with the key for the colors shown on the right.

**Figure 4.**
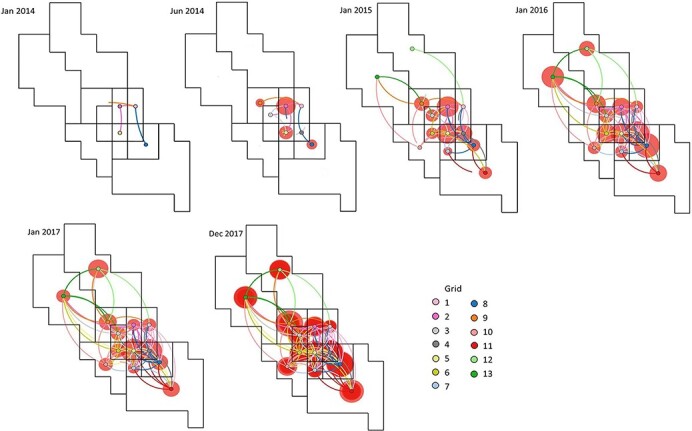
Maximum clade credibility (MCC) phylogeny of PRRSV sub-lineage 1A (L1A) diffusion in a swine-dense production area in the USA annotated with spatially discrete traits at intervals from 2014 to 2017. Lines between locations represent branches in the MCC tree representing the spatial transition. The branch color corresponds to the grid of destination of the branch. The diameters of red circles represent the number of sequences obtained at the location at the corresponding time period. In January 2014, we depict the early phase of spread, with L1A spreading from sectors 1 to 2, 2 to 5, and 1 to 8.

From the Skygrid analysis, we observed that the effective population size of PRRSV L1A in this region appeared to fluctuate seasonally, peaking during the summer months of 2015 and 2016 (Fig. 2). The estimated viral evolutionary rate was 1.8 × 10^‒2^ (95 per cent highest posterior density [HPD] 1.7 × 10^‒2^‒2.0 × 10^‒2^) per site per year. Moreover, the estimated time to the most recent common ancestor (TMRCA) of PRRSV L1A in this study region was the first quarter of 2013 (95 per cent HPD June 2012‒September 2013), indicating that all L1A diversity within this region stemmed from an ancestor existing around this time. This suggests a single introduction of this lineage to the region. Although this analysis was based on the ORF5 region only, we believe that an analysis of whole genome data would result in a TMRCA, as viral isolates classified into lineages based on ORF5 generally maintain their phylogenetic clades when using whole genome analysis (Schroeder et al., submitted). Between-sector transmission events occurred frequently within the study period; hence, the time-scaled phylogenetic tree does not show any distinct clustering based on sector (Fig. 3). These repeated transmissions to and from different sectors are also shown in Fig. 4, where we display the geographical context of the MCC phylogenetic tree at different time points during the study period.

### 3.2 Drivers of spatial diffusion

**Figure 5.**
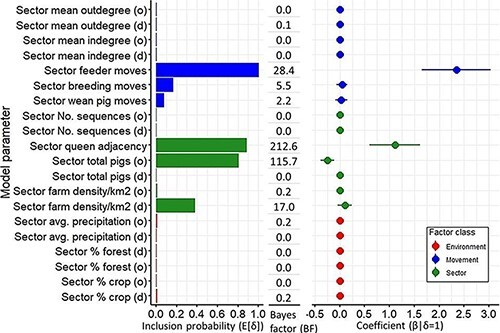
Predictors of the spatial diffusion of PRRSV sub-lineage 1A (L1A) between geographic sectors in a swine-dense production area in the USA. The left-hand panel shows the posterior inclusion probability for the different GLM predictors reflecting the frequency at which a predictor is included in the model, hence representing the support for the predictor’s association with the spatial transition of L1A. The right-hand panel shows the coefficient estimate of the model predictors on the spatial transition of L1A, which is a conditional effect size of each predictor on the rate of transition when the predictor is included in the model. Factors with BF > 6, are considered to have strong support for their inclusion in the model as factors influencing the spread of the virus. Factors with a BF >3 but <6, such as breeding movements, may have some influence on the viral diffusion trends observed. Environmental covariates are colored in red, sector-attributes in green, and movement-related factors in blue. Factors demarcated with (o) and (d) indicate factors pertaining to the origin or destination, respectively.

The geographical diffusion of PRRSV L1A in this area was significantly (BF > 6) influenced by animal movements, the spatial structure of the host population (sector adjacency), farm density in the destination sector, and pig population size in the origin sector. The movement of feeder pigs (from nursery to finishing farms or wean-to-finish to finishing farms [8–25 weeks]) greatly enhanced viral spread between sectors in the study area (posterior inclusion probability > 0.99 | BF = 28.4). Although the contribution of other animal movements to the viral transmission was minimal by comparison, breeding movements had a BF of 5.5, suggesting that they may also enhance the between-sector spread of L1A, to a much smaller extent. The effect of weaned pig movements was negligible (posterior inclusion probability <0.1 | BF = 2.2) (Fig. 5). The mean sector indegree and outdegree had no discernable effect on the rate at which PRRSV L1A spread across the region (posterior inclusion probability <0.1 | BF = 0). From additional analysis, all three movement types occurred with similar frequencies, and thus, frequency differences cannot explain the greater relative influence of feeder pig movements (Analysis of variance (ANOVA), Bonferroni adjusted *P*-value = 0.6). In addition, between-sector movements were not substantially correlated across type (Spearman correlation ‒0.2–0.4, *P*-values > 0.05) (Supplementary Table S1a and S1b and Fig. 2).

Viral spread was inversely associated with pig population size in the origin sector (posterior inclusion probability = 0.8 | BF = 115.7), whereas there was no evidence that population size in the destination sector influenced spread. Higher farm density in the destination sector also appeared to enhance viral spread (posterior inclusion probability = 0.37 | BF = 17). Higher transition rates were also observed between adjacent sectors (posterior inclusion probability = 0.88 | BF = 212.6) (Fig. 5). The number of sequences in either source or destination sectors and the farm density in the origin sector were not associated with observed viral transmission patterns (Fig. 5).

## 4. Discussion

By integrating viral sequence and animal movement data in Bayesian phylodynamic models, our research presents a novel approach for studying the spatial spread and evolutionary dynamics of PRRSV and other livestock diseases. The inclusion of empirical data on animal movements in phylodynamic models allows for more insightful inferences on disease spread, which can be useful in surveillance and management of disease outbreaks. In this analysis, we evaluated the impact of the movement of different age classes of pigs and found that the movement of feeder pigs (8–25 weeks old pigs moved from nurseries) was much more closely associated with patterns of spatial spread of PRRSV than other types of pig movements.

While animal movements in general have been highlighted as risk factors for PRRSV spread (Schulz et al., 2017; Ramírez et al., 2019; Silva et al., 2019; VanderWaal et al., 2020; Makau et al., 2021), we observed that some movements may contribute more to spatial spread than others. In this study population, the movements that contributed the most to geographical spread were feeder pig movements (Fig. 5). Alkhamis et al. (2016) and Jara et al. (2020) observed that sow farms were the likely origin of PRRSVs found in downstream farms in multi-site production systems in the USA, although Jara et al. (2020) also found evidence of PRRSV transmission between nurseries and finishers. However, those studies did not consider movements per se, but rather modeled how often the virus transitioned from one farm type to another without reference to spatial spread or empirical movement data on which farms were actually interconnected. Further, such discrete-trait phylodynamic analyses are susceptible to data imbalance and sampling bias, wherein the more common trait in the sampled sequences (i.e. sow farms) is likely to be identified as the ancestral population. In contrast, we empirically examined the role of specific movement types as related to sector-to-sector patterns of spatial spread. While there is no doubt in sow farm to nursery transmission, it did not appear to play a strong role in shaping spatial patterns of spread on the sector scale. Indeed, although we observed that the movement of weaned pigs (originating from sow farms) may have some effect (BF = 2.2) on wide-scale spread of PRRSV, the effect was much smaller than that of feeder pigs (originating from nurseries). We also observed a weak positive effect of breeding movements on the spatial spread of PRRSV in the study area. The movement of replacement gilts to sow farms is a potential avenue for disease spread. However, these movements conventionally include an acclimation phase during which pigs are vaccinated before being mixed with the breeding herd, thus minimizing risk of disease spread, but further research would elucidate this observation.

The importance of feeder pig movements in the spatial diffusion of PRRSV between sectors could be associated with several factors related to distinct characteristics of feeder movements and biosecurity of nursery farms. In most swine production systems, nurseries often receive weaned pigs from multiple sow farms, and the period of time that these pigs remain at the nursery coincides with waning of any maternal antibodies (Senn et al., 1999). In addition, biosecurity for nurseries is typically not as strict as for sow farms, making it easier for viruses to be introduced into and transmitted from the farm. For example, while transport trucks that serve sow farms are washed routinely, this is not always the case for feeder pig movements (personal communication). Thus, introduction of a virus in such an environment is likely to result in rapid amplification of the virus within the nursery and subsequent transportation of these pigs to finishing or wean-to-finish farms, culminating in dissemination of viruses between sectors in the study region.

In this study, there was a higher rate of viral transmission between adjacent sectors than non-adjacent sectors (queen adjacency, BF = 212), indicating that close geographical location was an important determinant for PRRSV spread. We cannot discriminate whether this is due to local-area spread between farms located close to borders or because pig movements and other mechanical routes of transmission (e.g. feed suppliers, waste management and rendering services, etc.) exhibit spatial clustering such that farms in neighboring sectors share service providers. Given the size of sectors, we believe the latter is more likely because most farms in two neighboring sectors are not within a reasonable distance (<10 km) for local-area spread (Mortensen et al., 2002; Larochelle, D’Allaire, and Magar 2003; Dee et al., 2009; Otake et al., 2010). Regardless of the mechanism behind transmission between adjacent sectors, our data suggest that between-sector spread among neighboring sectors can be expected.

The sector-level spatial resolution likely contributed to why we failed to find any effect of environmental factors often associated with local-area spread (Arruda et al., 2018a; Machado et al., 2019). As opposed to between-farm transmission at localized scales (among farms <10 km apart), environmental and land cover factors were not shown to play a significant role in the wide-scale spread of PRRSV at the broader geographical resolution of our study. While estimating secondary contacts and neighborhood risk attributes in areas nearby the farm can be beneficial in disease management, the limitations imposed by the sector-level approach utilized in this study obscured our ability to discern the influence of landscape factors that impact short-distance transmission. In addition, since the model setup did not allow for the inclusion of time-varying parameters, we only included a single value per sector for each environmental factor, including wind speed and ambient temperature, which were excluded from the model due to lack of variation. Averaging environmental data at the sector level across time led to the loss of fine-scale temporal and spatial variability in the data. For example, parameters such as tree cover and crop cover, previously reported as relevant for area spread of PRRSV (Arruda et al., 2018a; Machado et al., 2019), may have lost the inherent heterogeneity due to averaging over larger areas (sectors). It is also likely that by averaging network metrics (degree centrality) across the sectors, heterogeneity in the data was reduced; hence, the mean degree centrality in the model did not appear to influence between-sector viral dispersal. The notwithstanding limitation, we were able to identify other factors influencing L1A dynamics in the region at a broader scale and elucidate the long-distance spread of L1A in the study area. With a more detailed dataset, a modeling framework with higher spatial and temporal resolution could be used to identify other factors affecting the spatial spread and evolution of L1A in the area.

Sectors were not delineated based on known interaction patterns and farm connectivity in the region. Indeed, farm connectivity and clustering based on service providers such as feed suppliers, rendering, and waste management services could influence disease dissemination in the area. Using a different scale to create sectors may yield slightly different results. However, we believe the influence of factors with high BF and effect sizes, such as sector adjacency and movement of feeder pigs, would be robust to variations in sector boundaries. PRRSV L1A spread was also influenced by farm density (BF = 17), whereby high-density sectors were more likely to be the recipient of between-sector transmission events. Several studies have also highlighted the risk of disease spread and maintenance among farms in high-density areas (Firkins and Weigel 2004; Alkhamis et al., 2017, 2018; Arruda et al., 2017). Less intuitively, sectors with larger pig populations were less likely to be the source of PRRS spread to other sectors in the study area (BF = 115). Potentially, large populations could mean that pig movements can remain of shorter distance and thus not cross sector boundaries, although other unexplored factors could also drive this pattern. Of note, Pearson’s correlation between population size and farm density was 0.6.

The genetic diversity of the L1A viruses circulating in the region changed seasonally. In 2015, the genetic diversity increased rapidly in the spring season, peaking in the summer months and then declined. A similar trend was observed in 2016, although the change and the peak were slightly lower than in 2015. The periods of declining diversity seemed to align with ‘conventional’ PRRS season (November—May) during which newer PRRS outbreaks are typically reported in farms in many parts in the USA. (Tousignant et al., 2015; Arruda et al., 2018b). This inverse relationship could be occasioned by several factors. For instance, due to the reduced between-farm spread during the summer months, viruses documented during this time may represent farms with persistent infections (as opposed to new outbreaks). These persistent on-farm infections may diverge from the original outbreak strains through time. As such, the genetic distance between viruses isolated during this period would be larger than those isolated during the PRRS season. During the PRRS season, the rapid between-farm dissemination of prevalent outbreak strains may translate to a pattern whereby the viruses detected (the most prevalent strain) on different farms have little time to evolve between transmission events, thus leading to less apparent genetic diversity. We therefore postulate that these seasonal peaks in diversity could be driven by the factors mentioned above or other anthropomorphic factors. However, these trends require further investigation.

Although using self-reported data may be associated with some bias and data imbalance, this bias was considerably mitigated by several interventions employed in this study. The farms present in this study area belong to a very small number of production systems. Production systems have relatively consistent surveillance and diagnostic protocols across all farms managed by the system. The trust built through the long-standing working collaboration between veterinarians, production systems, and MSHMP reduced the likelihood of reporting biases by the production systems. Additionally, our continued adherence to confidentiality agreements reduced the likelihood of reporting bias.

Finally, partial immunity to closely related lineages/sub-lineages of PRRSV could influence the transmission dynamics of L1A in the region. Makau et al. (2021) observed that although prior exposure to non-L1A PRRSV reduced the likelihood of occurrence of L1A on farms in the study area, this association was not significant. However, further research is required to understand the evolutionary and dispersal dynamics of co-circulating strains in swine-dense production areas.

## 5. Conclusion

In this study, we highlight that while animal movements are generally important for the spread of PRRSV, the movement of feeder pigs is particularly critical for shaping the regional spread of PRRSV compared to other types of pig movements. As such, production systems should handle these types of movements more cautiously and increase disease surveillance and monitoring in nurseries. Producers could also consider standardizing the vaccination of wean pigs at the nurseries to harmonize immune profiles and institute measures to avoid the introduction of other viral variants to these farms This study also reiterates the relevance of host population connectivity in viral dissemination. Therefore, coordinated efforts to implement farm biosecurity measures for individual farms and their neighbors in a ‘localized’ area may help mitigate the spread of PRRS in the wider region. Finally, the inclusion of empirical animal movement data and the use of the GLM framework in BEAST enabled us to test novel hypotheses concerning the phylogeographic patterns of L1A spread. The incorporation of movement data into phylodynamic modeling, particularly stratified by the type of movement as done here, may advance our ability to discern the critical pathways of pathogen dispersal that can be targeted to mitigate the spatial spread of disease of both animal and human pathogens.

## Supplementary Material

veab060_Supp

## Data Availability

The data that support the findings of this study are not publicly available and are protected by confidentiality agreements.
