# Supplementary material for: Integrating animal movements with phylogeography to model the spread of PRRSV in the USA
Source: Virus Evol. 2021 Jul 15;7(2):veab060. doi: 10.1093/ve/veab060 (PMC8438914; doi:10.1093/ve/veab060)
Supplement: veab060_Supp [file veab060_supp.zip › Supplementary Figure 1.docx]

Supplementary figure 1: Demographic summary of farms and PRRS virus lineage 1A sequences in a swine dense production area in the U.S., between 2014 and 2017.The colors represent different farm types included in our study.


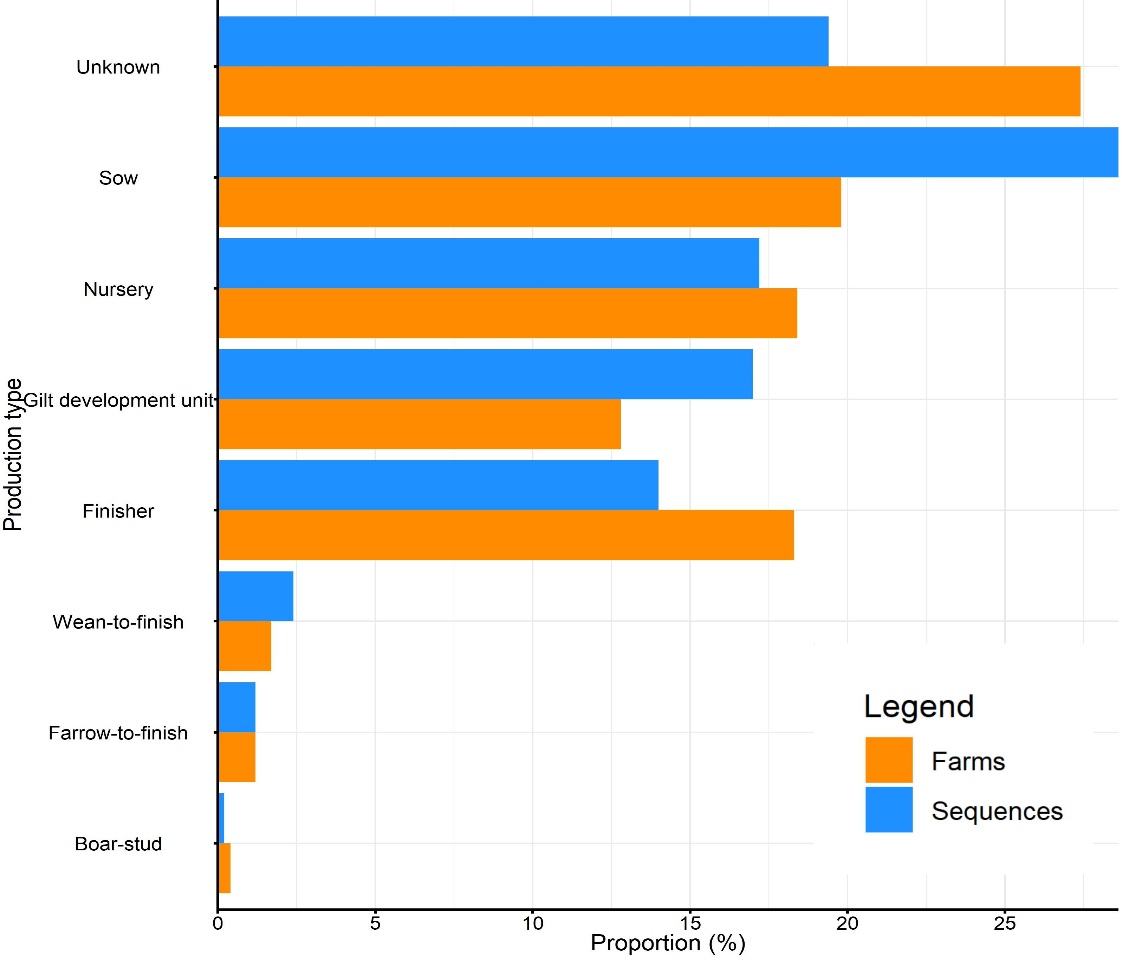


Sequences = 1514

Farms = 651

Sequences = 1433

Farms = 608

Sequences = 993

Farms = 516

Sequences = 984

Farms = 515

Sequences = 81

Farms with incomplete metadata = 43

Duplicate sequences = 440

Farms = 279

Recombinant sequences = 9

Farms = 1
