# Supplementary material for: Integrating animal movements with phylogeography to model the spread of PRRSV in the USA
Source: Virus Evol. 2021 Jul 15;7(2):veab060. doi: 10.1093/ve/veab060 (PMC8438914; doi:10.1093/ve/veab060)

Supplementary figure 2: A network of between-sector pig movements in the study region. Edges are weighted by the log number of movements and colored by type of movement.
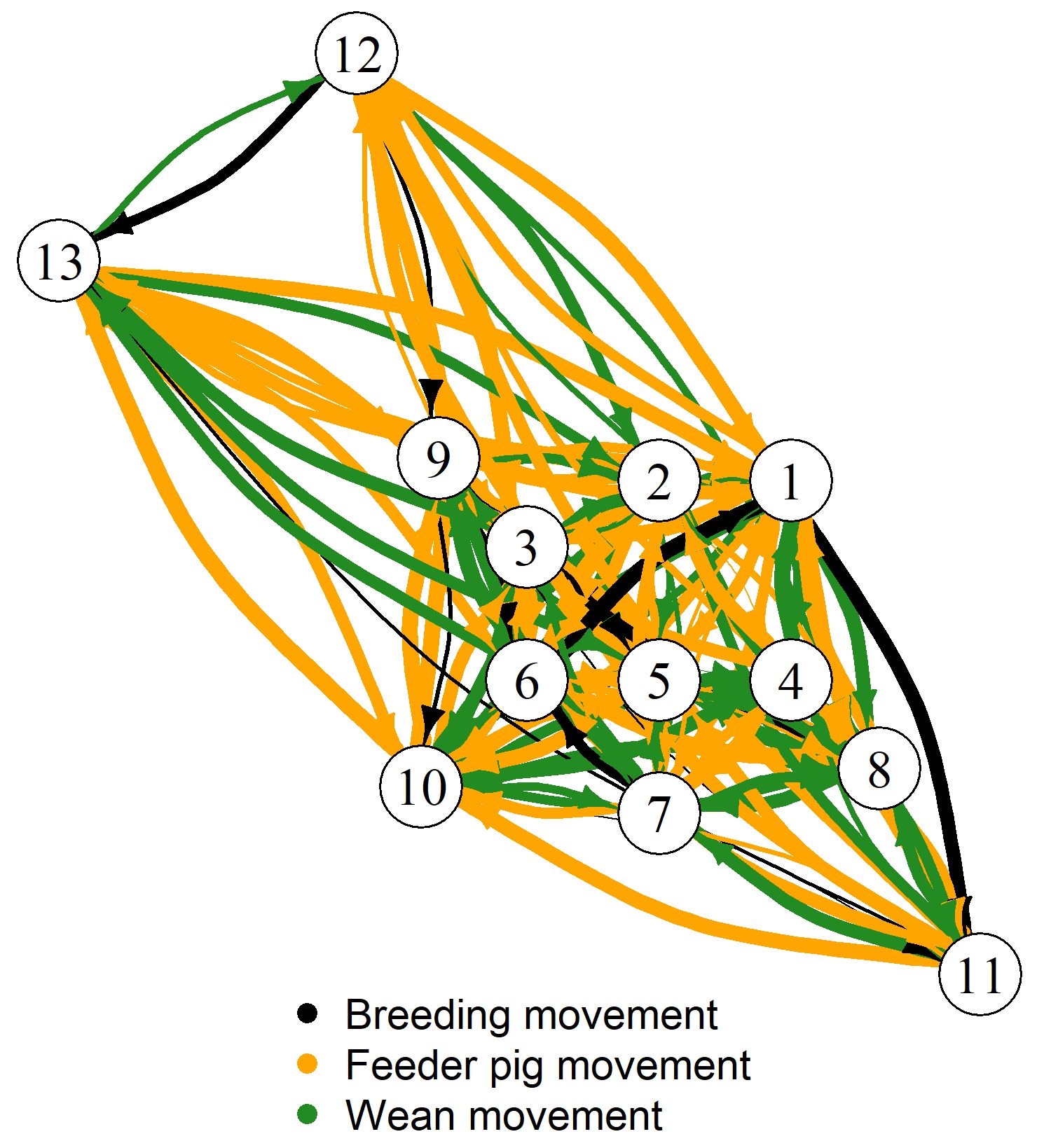

Supplement: veab060_Supp [file veab060_supp.zip › Supplementary Figure 2.docx]
