# Supplementary material for: Integrating animal movements with phylogeography to model the spread of PRRSV in the USA
Source: Virus Evol. 2021 Jul 15;7(2):veab060. doi: 10.1093/ve/veab060 (PMC8438914; doi:10.1093/ve/veab060)
Supplement: veab060_Supp [file veab060_supp.zip › Supplementary Table 1.docx]

| Type of movement | Mean | Std. Dev | ANOVA p value |
| --- | --- | --- | --- |
| Breeding movements | 651.67 | 905.26 | 0.64 |
| Feeder pig movements | 752.47 | 1023.01 |  |
| Wean pig movements | 640.62 | 922.09 |  |

Supplementary table 1

a: Summary of ANOVA analysis of frequency of between and within sector pig movements by type of movement in a swine dense production are in the U.S. *Bonferroni adjustment was used for the P value.*

b: Spearman’s correlations of model predictors used in the GLM model discrete space phylodynamic model for L1A spread in a swine dense production area in the U.S.

|  |  | P values | | | | | | | | | |
| --- | --- | --- | --- | --- | --- | --- | --- | --- | --- | --- | --- |
|  |  | 1 | 2 | 3 | 4 | 5 | 6 | 7 | 8 | 9 | 10 |
| 1 | Avg. precipitation |  | 0.61 | 0.10 | 0.73 | 0.73 | 0.73 | 0.61 | 0.73 | 0.22 | 1.00 |
| 2 | Total pigs | 0.16 |  | 0.48 | 0.10 | 0.54 | 0.46 | 0.75 | 0.12 | 0.19 | 0.16 |
| 3 | Forest % cover | -0.47 | -0.21 |  | 0.04 | 0.51 | 0.23 | 0.25 | 0.13 | 0.97 | 0.82 |
| 4 | Crop % cover | -0.10 | 0.48 | -0.57 |  | 1.00 | 0.22 | 0.01 | 0.63 | 0.46 | 0.08 |
| 5 | Mean indegree | -0.10 | -0.19 | 0.20 | 0.00 |  | 0.18 | 0.30 | 0.29 | 0.60 | 0.28 |
| 6 | Mean outdegree | 0.10 | -0.23 | 0.36 | -0.36 | 0.40 |  | 0.09 | 0.22 | 0.96 | 0.41 |
| 7 | Breeding moves | 0.16 | -0.10 | 0.35 | -0.70 | 0.31 | 0.49 |  | 0.16 | 0.49 | 0.05 |
| 8 | Wean moves | -0.10 | 0.46 | 0.44 | -0.15 | 0.32 | 0.37 | 0.42 |  | 0.19 | 0.99 |
| 9 | Feeder moves | 0.37 | 0.39 | -0.01 | 0.23 | 0.16 | -0.02 | -0.21 | 0.39 |  | 0.07 |
| 10 | Farm density | 0.00 | 0.41 | 0.07 | 0.50 | -0.32 | -0.25 | -0.56 | -0.01 | 0.53 |  |
|  |  | Correlation coefficients | | | | | | | | | |
